# Clinical, Ethical, and Legal Considerations Raised by Self-Reported Genital Mutilation Following Voluntary Cosmetic Labiaplasty

**DOI:** 10.1007/s10508-024-03058-2

**Published:** 2024-12-27

**Authors:** Tania Metaxas, Brian D. Earp, Dina Bader, Sotoudeh Ghasemi, Milena Solari, Jasmine Abdulcadir

**Affiliations:** 1 Gynecology Division, Department of Pediatrics, Obstetrics and Gynecology, Geneva University Hospitals, Boulevard de la Cluse 30, 1205 Geneva, Switzerland; 2 Centre for Biomedical Ethics, Yong Loo Lin School of Medicine, National University of Singapore, Queenstown, Singapore; 3 Uehiro Oxford Institute, University of Oxford, Oxford, UK; 4 Independent Expert, Geneva, Switzerland; 5 Faculty of Medicine, University of Geneva, Geneva, Switzerland

**Keywords:** Labiaplasty, Female genital cosmetic surgery, Female genital mutilation, Female genital mutilation/cutting, Informed consent

## Abstract

An increasing number of women are undergoing female genital cosmetic surgery (FGCS). Labiaplasty, the most commonly performed FGCS, consists of a surgical procedure to decrease the inner labia size so that no or less tissue protrudes beyond the outer labia. Anatomically, it is similar to female genital mutilation/cutting (FGM/C) Type 2a. Thus, what are the differences and similarities between FGCS and FGM/C? Even though FGCS is not recommended by most scientific societies, it is considered legal, contrary to FGM/C. Most women seeking FGCS have physiologic (neither diseased nor anatomically atypical) genitalia and are reassured by counseling. We recommend counseling, history taking, screening for relevant conditions, and, if surgery is pursued, medical care by a specialist. We present the cases of three patients who reported feeling genitally mutilated after having willingly undergone FGCS. This feeling led these women to seek care at an outpatient clinic that receives migrants, refugees, second-generation, or naturalized patients originating mainly from African countries having experienced FGM/C. We discuss clinical implications, health insurance coverage, legal, ethical, and social implications. Multiple unresolved issues must be carefully addressed by scientific societies, legislators, and anti-FGM/C advocates to ensure equal treatment of all individuals in relation to genital cutting or surgery. Genital modifications experienced as harmful, or that are performed without informed consent, should be studied in relationship to one another and evaluated with consistent principles, regardless of the skin color of the individuals concerned, their cultural, ethnic, or religious background, or the name given to the genital modification they underwent.

## Introduction

The World Health Organization (WHO) defines “female genital mutilation” (FGM) as any cutting or modification of the external female genitalia (i.e., vulva) done for “non-medical reasons” (WHO, 2023). Taken literally, this official and widely used definition can imply that identical modifications of the vulva resulting in the same physical appearance could be deemed mutilating or non-mutilating based solely upon the perceived “reasons” for which the act was undertaken, e.g., therapeutic, cosmetic, “cultural,” or ritual/religious. However, official definitions notwithstanding, whether a given genital modification should indeed be regarded as mutilating is plausibly not (solely) a question of the real or perceived *reasons* for which the modification was undertaken, which are, in any case, not necessarily provable and may also vary depending on the context or community. Rather, it seems it should also depend on the *consequences* of the modification (including the resulting appearance, potential complications, and so on) and how these consequences are experienced or interpreted by the affected individual (Earp, 2019). Here, we present the cases of three women who considered themselves to have been genitally mutilated after willingly undergoing labiaplasty, a type of female genital cosmetic surgery (FGCS) that is commonly practiced in Western countries, and which is generally considered acceptable and legal.

What, then, makes a procedure a “mutilation”? One potential way to distinguish FGM (also known as female genital mutilation/cutting, or FGM/C) and Western-associated FGCS is that the latter are characteristically performed by licensed surgeons, whereas the former are more often performed by “traditional” cutters, who may or may not have medical training. However, given the increasing medicalization of FGM/C, as discussed below, the WHO definition has been expanded to cover fully “medicalized” forms of the practice (i.e., including forms carried out hygienically by trained healthcare providers, similar to most FGCS). Therefore, it remains unclear what principled difference there is between FGCS such as labiaplasty and so-called medicalized FGM/C: both are performed by healthcare providers to alter non-diseased vulvar anatomy, without a clear medical justification (Shahvisi et al., 2024).

“Medicalized FGM/C” is increasingly common in many high-FGM/C prevalence countries (Van Eekert et al., 2024) and is said to violate both medical ethics and human rights (The Lancet, 2024). It is also a criminal act in many countries, with various jurisdictions drawing directly on the WHO definition to specify the crime (O’Neill et al., 2020). Given the considerable stakes involved, not only for individuals who risk being harmed by such procedures, but also for healthcare providers, who might face criminal charges if accused—rightly or wrongly—of performing FGM/C, it is necessary to be clear and unambiguous about the precise basis for distinguishing procedures that are considered to be FGM/C from other, potentially similar procedures that, unlike FGM/C, are not alleged to violate medical ethics, human rights, or criminal law (O’Neill et al., 2020).

One potential candidate for distinguishing between unethical, illegal, or human rights-violating modifications of the vulva from physically similar yet presumptively permissible modifications, would be whether to not the individual *consented* to the modification, thereby waiving their moral right to bodily non-interference for purposes of the procedure (Townsend & Earp, in press). However, neither the WHO definition of FGM/C (whether “medicalized” or “unmedicalized”) nor at least some national laws such as the Swiss anti-FGM/C Act, discussed later, refer to consent (or any related concept such as age/maturity, legal majority/minority status, and choice/voluntariness) as a criterion for distinguishing FGM/C from anatomically comparable procedures not considered to be FGM/C, such as those falling under the label of FGCS.

Instead, as mentioned previously, the WHO definition appeals exclusively to “non-medical reasons” to distinguish between FGM/C and non-FGM/C and these reasons themselves are not defined. This could suggest that even if an alteration to the vulva was done by a competent surgeon at the explicit request—and with the fully informed consent—of the individual, it could still technically count as “FGM/C” on the WHO definition, unless done for unspecified “medical reasons.” And yet, resting such an important and consequential distinction on this single phrase could be problematic. First, although an explicit “medical reason” might well be provided in certain cases, this alone does not guarantee it is a *good* or *sufficient* medical reason for ethically proceeding with a genital procedure. Second, there may be uncertainty around what makes a given reason a “medical” reason in the first place, i.e., as opposed to some other sort of reason (such as a “cultural” or “cosmetic” reason).^1^ And third, there may sometimes be a *mix* of medical and non-medical reasons at play in motivating certain genital procedures, without it being clear which type of reason predominates (Walden et al., 2024).

However, suppose it were simply stipulated that a given vulva modification was done for all and only “non-medical reasons.” Still, it would not be obvious why this fact alone should render the modification a “mutilation.” To illustrate, suppose a given procedure is done for *clearly* non-medical reasons (for example, a voluntary labiaplasty requested in adulthood for purely aesthetic reasons, with no medical justification whatsoever). Even if the operation is successful and the patient is entirely satisfied with the result, has she nevertheless experienced a form of genital “mutilation”?

Now suppose an anatomically equivalent procedure is done for clearly *medical* reasons (for example, to remove malignant tissue), and yet the operation leaves a highly disfigured appearance, and causes severe and persistent functional difficulties. Could the patient not reasonably regard herself to have been—albeit inadvertently—genitally mutilated in such a case?

To summarize, if one relies on the current official definition of the WHO, vulva modifications could in principle be categorized as either mutilating or non-mutilating based upon the perceived “reasons” for which the act was undertaken, irrespective of whether the procedure was carried out by a qualified surgeon under hygienic conditions, rather than based on several criteria that might intuitively seem more reasonable or relevant, such as whether the individual consented to the procedure or considers the outcome of the act to be an enhancement or improvement of the body versus a harm or disfigurement.

These definitional matters are not just theoretical. In this paper, we present the cases of three women (two Swiss, one French) who consulted a specialized outpatient clinic for women and girls with FGM/C because they felt, quote, “mutilated, like the women who live in countries where FGM/C is traditional” after having willingly undergone FGCS to reduce the size of their inner labia (i.e., labiaplasty). One patient had previously consulted another gynecologist-sexologist, another one a plastic surgeon to be “repaired,” and the last one an emergency department. All of them declared themselves to be “mutilated” to their respective doctors before consulting the clinic.

The FGM/C clinic was founded in 2010 in the public University Hospitals of Geneva, Switzerland, to meet the healthcare needs of immigrated women and girls from primarily African countries where female genital modifications are highly prevalent and considered to be customary. FGM/C prevalence among women and girls is estimated to be 99% in Somalia, 95% in Guinea, 90% in Djibouti, 89% in Mali, 87% in Sudan, 87% in Egypt, and 83% in Eritrea (UNICEF, 2024). It is therefore noteworthy that the three women whose cases we discuss in this paper, among a few others who did not consent for their cases to be published, were all born and raised (with recent ancestry) in European countries. Two of them were racialized as white, one as black; however, the latter patient did not have recent ancestry in any of the countries where FGM/C is prevalent or customary.

Female genital cosmetic surgery (FGCS) is a broad term used to refer collectively to multiple surgical procedures aimed at changing the structure and appearance of a person’s vulva or vagina, characteristically in the absence of a relevant physical disease. These include labiaplasty, clitoral hood reduction, hymenoplasty, labia majora/outer labia augmentation, vaginoplasty, cosmetic clitoridectomy, and G-spot amplification (ACOG, 2020). The most commonly performed FGCS is labiaplasty, mainly practiced by gynecologists and plastic surgeons (Simonis, 2015), with the number of requests consistently growing: for example, a 217.3% increase was observed over a five-year period in the USA (The American Society for Aesthetic Plastic Surgery, 2015), while a corresponding 5-year increase worldwide has been estimated at 46.3% (with the total number of labiaplasties estimated as 194,086 worldwide in 2022) (International Society of Aesthetic Plastic Surgery, 2022). Such increases may be due to multiple factors, including advertising by cosmetic surgeons referring to and/or heightening bodily and sexual insecurities; a lack of knowledge of the physiological spectrum of the labia; current Western beauty norms favoring a vulva with a prepubertal (“juvenile”) appearance; and an increase of hair removal and exposure to digitally modified or pornographic vulvar images available on the Internet (Simonis, 2015).

For several decades now, scholars across multiple disciplines have been debating the similarities and differences between FGCS and FGM/C (Bader, 2016; Boddy, 2020; Davis, 2002; Earp et al., 2023a; Gaffney-Rhys, 2021; Shahvisi, 2021). In both types of intervention, non-pathological genital anatomy is cut, removed, or otherwise altered in the absence of a clear physical health indication. Both sets of procedures have been medicalized in many contexts (that is, performed by a trained healthcare professional whether in a public or private clinic, at home or elsewhere), although not (yet) to the same extent (Brussels Collaboration on Bodily Integrity, 2019; Van Eekert et al., 2024). For example, so-called medicalized FGM/C has been reported in up to 78.4% of cases in Egypt (Shell-Duncan, 2018). Indeed, some practitioners of “FGM/C” in Egypt have taken to simply calling the same procedures “cosmetic” genital surgeries, since modifications falling under the latter label—in Western countries and in Egypt—are not regarded as illegal or as violations of human rights (El-Gibaly et al., 2019; O’Neill et al., 2020).

The motivations of health professionals carrying out FGM/C include the belief that performing FGM/C themselves would be less harmful for girls or women than by a traditional practitioner (the so-called harm reduction perspective); followed by the belief that the practice was justified for cultural or religious reasons; for financial gains; and responding to community requests or pressure (Doucet et al., 2017). The resulting genital morphologies after some types of FGM/C and FGCS can be similar or even practically indistinguishable (Shahvisi, 2021).

While it is true that practices defined as FGM/C are more commonly performed on individuals under the age of 18 (for various reasons that may differ from group to group, such as seeking social acceptance, marriageability, pre-marital virginity, purity, cleanness, and beauty; WHO, 2008), FGCS is *also* performed on legal minors (see statistics below) (Jothilakshmi et al., 2009; Umbricht-Sprüngli & Gsell, 2016); while at the same time, what is characterized as “FGM/C” is often culturally considered to be the very act that is necessary for attaining adult status and recognition in the community, complicating the question of what it means to consent in different socio-cultural contexts. Even so, as mentioned, the WHO definition of FGM/C does not refer to consent (for critical discussion, see Earp & Johnsdotter, 2021), and neither does the Swiss criminal code (Swiss Confederation, 2011).

**Fig. 1:**
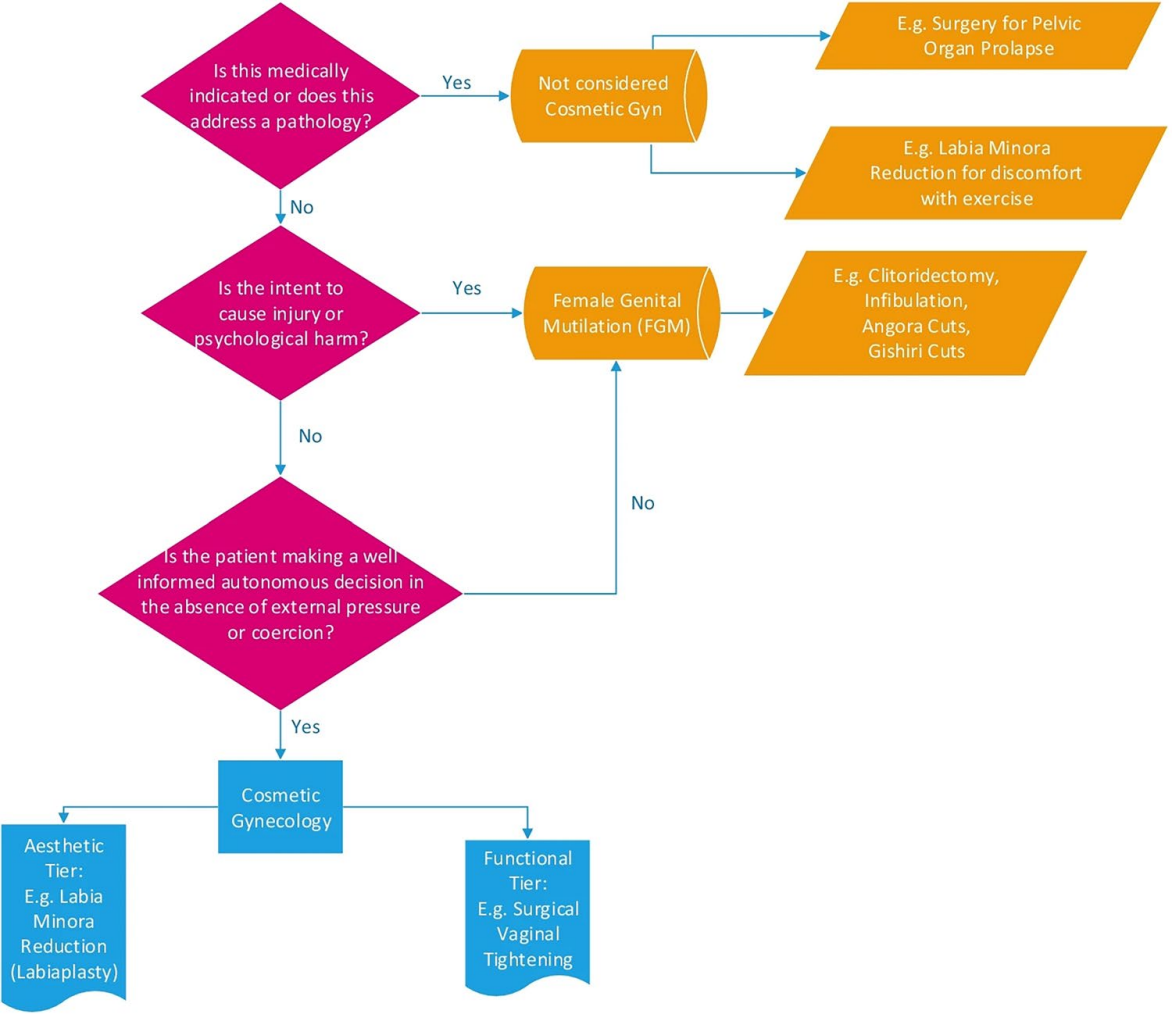
Is this cosmetic gynecology? (Joint Writing Group of the International Urogynecological Association & the American Urogynecologic Society, 2022)

The situation has become sufficiently confusing that a joint report on the question has recently been published by a Joint Writing Group of the International Urogynecological Association and the American Urogynecological Society (2022), hereafter abbreviated as IUGA/AUGS. The report, complete with a flowchart (Fig. 1), guides the reader through various terminologies and distinctions meant to “separate” cosmetic gynecology procedures from FGM/C. It does so by asking whether the genital procedure was medically indicated, intended to cause harm, and consented. But these criteria do not, in practice, allow one to draw a clear and determinate line between the allegedly unacceptable forms of vulva modification (“FGM/C”) and the allegedly acceptable forms (“FGCS”). This is due to (a) a lack of clarity around what counts as an appropriate “medical indication” for modifications of non-diseased genital tissues (Walden et al., 2024); (b) the fact that most “FGM/C” is not intended to cause net harm to the individual, but rather is expected to bring overall benefits (e.g., in terms of social acceptance, marriageability, and so on; for discussion, see Rogers, 2009), and (c) the above-stated lack of a consent criterion on the WHO definition. Further limitations of the definition are summarized in Table 1 (see also Shahvisi et al., 2024).

**Table 1:** Shortcomings of the WHO definition of female genital mutilation. Adapted from Shahvisi et al. (2024)

The definition…	(a) does not specify whether “female genitalia” includes the genitalia of females with differences/variations of sex development or intersex traits, an increasingly relevant concern as non-therapeutic intersex surgeries are being legally contested in a growing number of countries (Danon et al., 2023);
	(b) is silent on the question of whether others besides (non-intersex) females are definitionally mutilated by “all procedures that involve partial or total removal of [their] external genitalia, or other injury to [their] genital organs for non-medical reasons” (for example, intersex children born with penile variations, as well as non-intersex males including both cisgender boys/men and transgender girls/women, all of whom are at risk of such procedures, often without their consent, as in the case of newborn penile circumcision);
	(c) does not distinguish between voluntary and non-voluntary or forced genital procedures;
	(d) does not clarify the meaning or scope of the term “non-medical”—despite this being the sole criterion to distinguish “mutilating” forms of female genital cutting or modification from non-mutilating forms;
	(e) does not say how strong or weak a “medical reason” must be for an otherwise identical genital procedure or morphology to switch from being an instance of mutilation to one of non-mutilation (thereby potentially incentivizing proponents of contested genital procedures to seek out medical reasons or rationales for doing them) (Earp et al., 2023b);
	(f) does not give advice on how to classify genital practices done for a *mix* of “medical” and “non-medical” reasons (Hellsten, 2004)

Despite these shortcomings, the official WHO definition remains influential in shaping empirical research in science and medicine, also being incorporated into laws and policies, determining, for example, which acts—and therefore which persons, both requesting and performing such acts—are criminalized versus treated as legal (Bootwala, 2023).

## Labial Modification: Female Genital Mutilation/Cutting or Female Genital Cosmetic Surgery?

Where genital cutting of girls is prevalent and considered customary (FGM/C), it is usually performed before the age of 15, and, depending on the type, degree of medicalization, and other factors, can cause several psychosexual and genitourinary complications (UNICEF, 2022). WHO FGM/C Type 2a is defined as the total or partial removal of the inner labia (WHO, 2018). Type 2b also involves cutting of the prepuce and/or glans of the clitoris (WHO, 2018).

Labiaplasty (FGCS) is a surgical procedure to decrease the size of the inner labia so that no or less tissue protrudes from the outer labia (i.e., anatomically similar to FGM/C Type 2a) (Singh, 2020). It can be performed together with a cutting, reduction, or lifting of the clitoral prepuce (similar to Type 2b) (American Society of Plastic Surgeons, 2020a). Although more often performed in adulthood, at least 267 labiaplasties were performed on girls aged under 14 years old in the UK between 2008 and 2012 (British Society for Paediatric & Adolescent Gynaecology, 2013). In the USA, in fact, the highest rate of labiaplasties is observed among adolescent girls and young women, with nearly 20% of all such procedures between 2018 and 2019 being performed on girls younger than 18 years old (Luchristt et al., 2022). The rate of labiaplasties involving also clitoral prepuce/glans procedures is unavailable—there is one case report about a cosmetic clitoridectomy in a 33-year-old woman who had already had a labiaplasty (Veale & Daniels, 2012).

It is often assumed that FGM/C has higher complication rates than FGCS. However, if one controls for procedure type, degree of medicalization, and skill of the practitioner, all of which vary considerably both within and between the two sets of procedures, with a substantial amount of material overlap, such an assumption is no longer tenable. Moreover, although FGM/C-related complications are often emphasized in media reports and advocacy materials (PPAN, 2012), FGCS-related complications are not as widely spoken about, which may contribute to the (mis)impression that the latter set of procedures is inherently less risky or consequential.

Complication rates for labiaplasty reported among women vary between 4 to 18% (Boddy, 2020). Common reported postoperative complications include dehiscence, hematoma, flap necrosis, visible scarring, asymmetry, frayed wound edges, contour defect, gaps, over-resection, and under-section (Motakef et al., 2015); pain, infection, and decreased sexual arousal (Schober et al., 2010); and dissatisfaction with the new genital anatomy (Gress, 2021). This may result, for instance, from postoperative excessive tissue around and above the clitoral hood (called the small penis deformity), with the need for corrective surgery (Gress, 2021).

Gress (2021), a German plastic surgeon, reported having performed 702 reconstructions to rectify postoperative deformities after inner labia reductions between 2014 and 2020. Post-primary labiaplasty, 98% of his patients who sought reconstructive surgery felt psychologically impaired by the appearance of their inner labia, 77% had functional issues, 76% experienced pain (e.g., during sexual intercourse), and 14% had to seek help with a psychologist or psychiatrist. Out of patients who needed inner labia reconstruction, 43% underwent multiple corrective surgeries. No data exist on psychosexual complications.

Another way to see the overlap between FGM/C and FGCS is to consider the labial vasculature and nerve tissues involved (Cao et al., 2021), and the technical similarity of surgical means of repairing associated vulvar damage in either case. For example, a recent article describes reconstructive surgery for amputated labia minora using a labia majora flap, where the procedure is said to be applicable after a history of either FGM/C Type 2a or a labiaplasty with undesired outcomes (Awwad et al., 2024).

## Clinical Presentations

In this case series, we present three patients who spontaneously consulted or were referred to Geneva University Hospitals’ specialist FGM/C clinic due to a feeling of “being mutilated” following cosmetic labiaplasty. As they explained, this meant a feeling of having been damaged, harmed, and made to suffer physically and psychosexually because an essential part of their body had been removed in a way they had not imagined and therefore did not feel they were able to validly consent to.

The women also felt they had been made uglier in the sense that they regarded their vulvas as disfigured following the procedure (all of them reported that their labia were “normal” before surgery; we could obtain the preoperative picture for only one of them, who had a physiologic vulva). All signed an informed written consent for this case series and the publication of their pictures. We describe the psychophysical and psychosexual complications following labiaplasty as well as the psychophysical care offered. Two women received conservative psychosexual care while one received psychosexual care and reconstructive surgery. We discuss the clinical, legal, social, and ethical issues raised by these three cases.

## Case 1

**Fig. 2:**
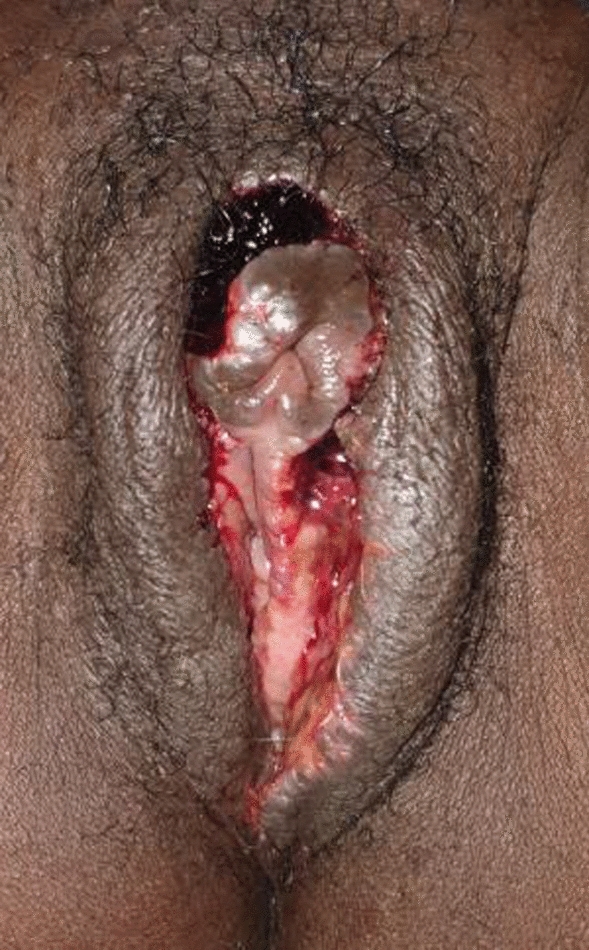
Patient 1, 14 days after labiaplasty and clitoral lifting

**Fig. 3:**
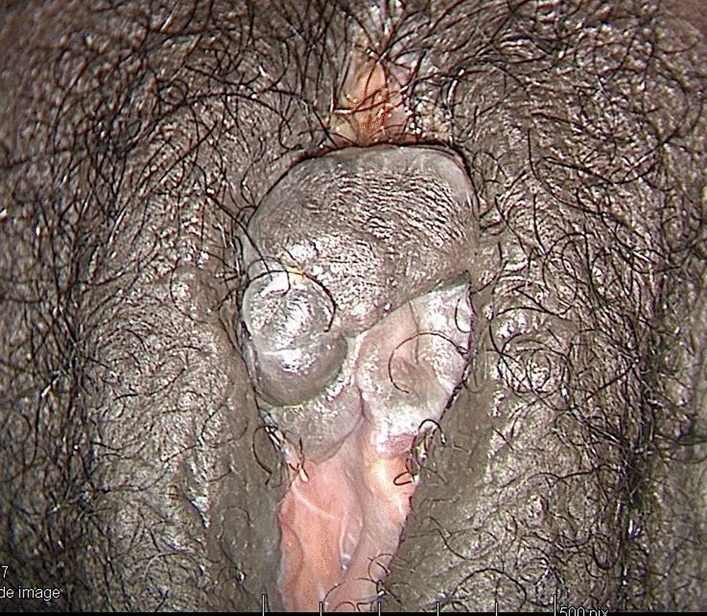
Patient 1, conservative treatment

A 38-year-old French woman was referred to the FGM/C outpatient clinic after consulting the ObGyn emergency department for an uninfected wound dehiscence of the inner labia and clitoral prepuce, 14 days after undergoing labiaplasty and clitoral lifting in Morocco (Fig. 2). FGCS had been done at the same time as an abdominal liposuction and mammary prosthesis replacement. The dehiscence was successfully managed conservatively with showers, hyaluronic acid, and silver sulfadiazine cream and dressings (Fig. 3).

**Fig. 4:**
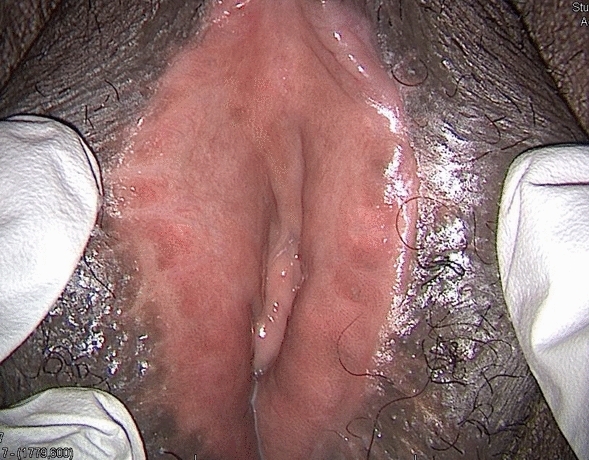
Patient 1, four months after labiaplasty

**Fig. 5:**
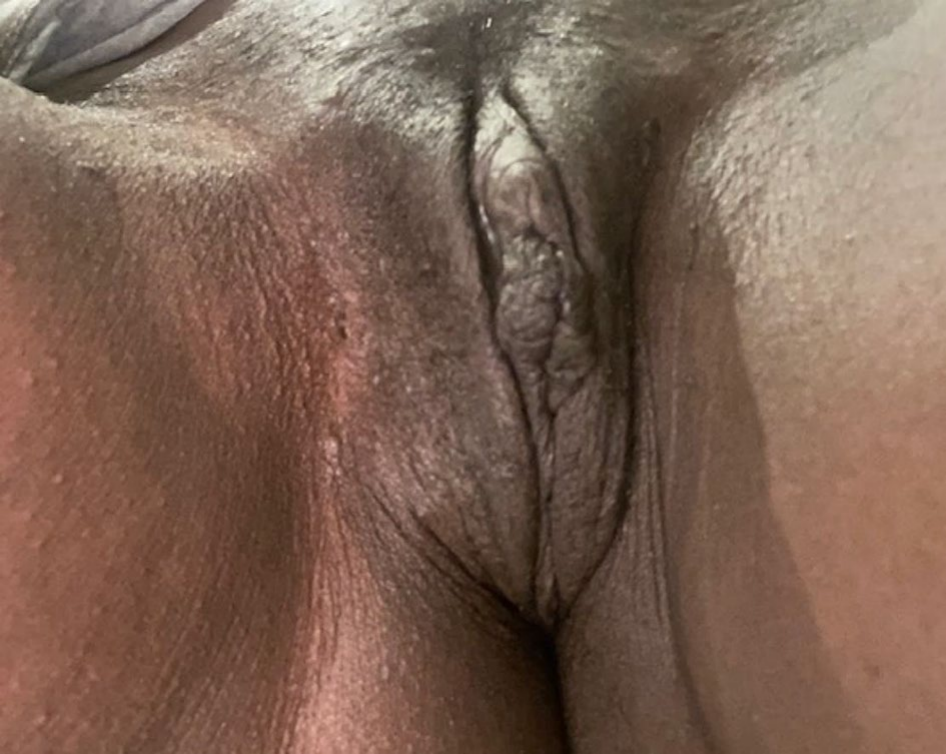
Patient 1, preoperative vulvar appearance

**Fig. 6:**
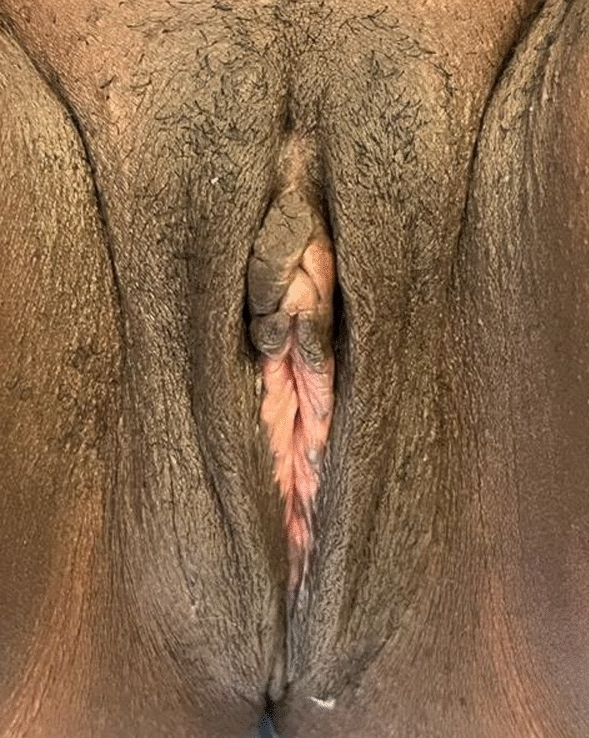
Patient 1, almost 5 months after labiaplasty and clitoral lifting. Conservative management of the dehiscence

Four months post-op (Fig. 4), the patient was dissatisfied with her genital image and missed her pre-op vulvar appearance (Fig. 5) because the glans was “too protuberant” with a remaining foreskin asymmetry in favor of the right side. She also disliked the fact that she “did not have any inner labia.” She resumed sexual intercourse but suffered from superficial dyspareunia. She was prescribed physiotherapy, local estradiol, and testosterone compounded cream (0.01–0.1%) (Burrows, 2013). From the beginning, she was offered psychosexual counseling covering vulva and clitoris anatomy and physiology, sexual response, and body image. The healing of her genitals was regularly shown to the patient. 145 days post-labiaplasty (Fig. 6), her dyspareunia was gradually significantly decreasing as was the dissatisfaction with her genitals.

During psychosexual counseling, a past history of sexual violence was disclosed. The patient was referred to our sex-therapist psychologist, whom she did not, however, consult. The patient declared deeply regretting the labiaplasty and felt like her labia were “normal” and “more beautiful” before surgery. She admitted having trivialized the scope of the vulvar surgery and potential postoperative complications. She was nonetheless satisfied with the other aesthetic procedures on her abdomen and breasts. She was not willing to undergo any further vulvar surgical reconstruction.

## Case 2

A 31-year-old nulligravida Swiss patient had a cycling accident at age 17, with a tear of her left inner labium. The labium was immediately sutured in the emergency room under general anesthesia with subsequent wound dehiscence. Since then the patient suffered from spontaneous and provoked chronic pain on the inner left labium, as well as superficial dyspareunia and itching. She was also diagnosed with post-traumatic stress disorder (PTSD) related to the accident. Her medical history established that she also suffered sexual and intimate partner violence before and after the surgery.

After ten years of symptoms, vulvar surgery revision of the left labium was performed in Switzerland. She however suffered from a new postcoital wound dehiscence three months later. A new surgery was performed with reduction of the failed labium and a contralateral labiaplasty to obtain inner labia symmetry.

**Fig. 7:**
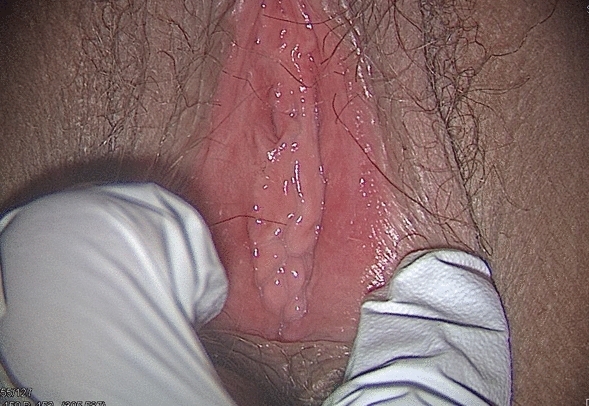
Patient 2, 2 years after revision surgery

In November 2019 (two years post-revision surgery) the patient asked her sexologist gynecologist to consult the FGM/C outpatient clinic to address her feeling of having been genitally mutilated, particularly on the healthy labium that was reduced for purposes of symmetry. She was already treated for vulvodynia with local and systemic treatments with a good management of the pain and only occasional feeling of itching. At the clinical exam, her vulva presented an absence of inner labia and no other medical conditions such as Lichen Sclerosus (Fig. 7).

The patient did not want a surgical reconstruction but wanted to better accept her genitals, to decrease her feeling of being mutilated, and to reduce her itching symptoms on the labia that appeared during stressful moments. She received counseling on anatomy and physiology of the vulva including oral and written information on the labia and vulva diversity, FGM/C, labiaplasty, and was referred to a psychologist trained in trauma. The itching was not found to be associated with any dermatologic or other vulvar condition and was managed with fatty ointments and counseling.

The patient was followed for two years before being referred again to her sexologist gynecologist. She expressed having less labial pain, less superficial dyspareunia and suffered from itching only during stressful days. She reported greater awareness and acceptance of her anatomy but still defined herself as mutilated. She did not think that inner labia reconstruction would improve her well-being or self-acceptance.

## Case 3

A nulligravida 44-year-old Swiss woman with a past history of heroin addiction treated by substitution therapy (in which a substitute substance is prescribed to build the therapeutic alliance and decrease the drug’s high-risk use) underwent a cosmetic wedge labiaplasty in Switzerland, followed two weeks later by corrective surgery due to wound failure on the left inner labium. She consulted the FGM/C clinic and a private plastic surgeon six months after this corrective surgery. The woman declared that she deeply regretted the surgery, disliked the look and indurated sensation of the skin of her vulva while feeling obsessed with and disgusted by the result.

**Fig. 8:**
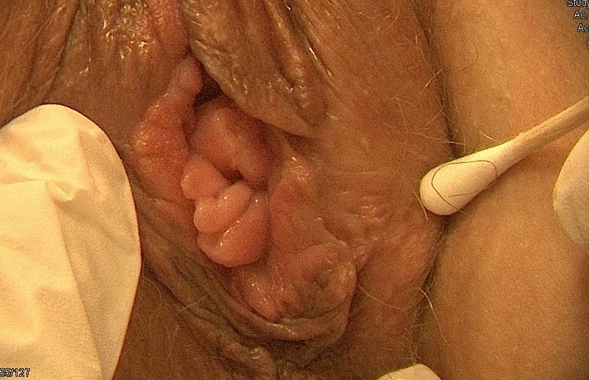
Patient 3, 6 months after revision surgery

**Fig. 9:**
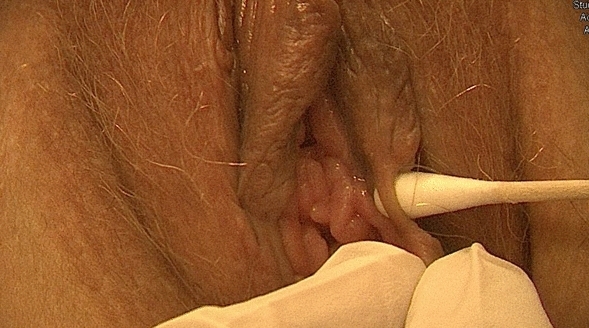
Patient 3, 6 months after first revision surgery

Multiple labial scars and an extro-flexed hymen were observed during the physical examination (Figs. 8 and 9). Counseling, physiotherapy, and psychological support were offered to the patient.

**Fig. 10:**
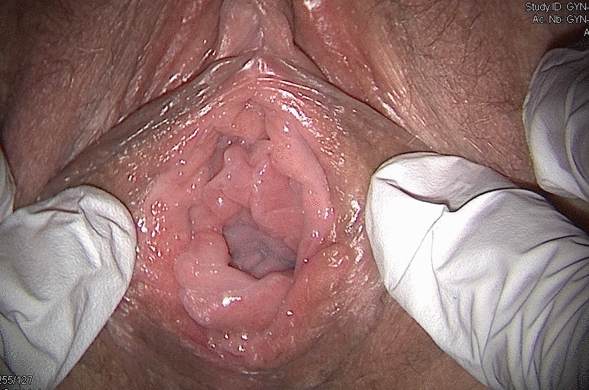
Patient 3, 5 months after reconstructive surgery

She had not resumed sexual intercourse since the labiaplasty due to negative genital self-image. She could nevertheless experience self-pleasure and orgasm through masturbation. The patient experienced a relapse of her drug addiction shortly followed by suicidal thoughts that led to a psychiatric hospitalization. She underwent reconstructive surgery of her labia with a plastic surgeon 20 months after labiaplasty and once her mental health was cared for and improved (Fig. 10). While continuing to regret the first labial intervention and her pre-op genital appearance, she was satisfied with the reconstructive surgery and the care received.

## Discussion

The three cases we have presented show that labiaplasty can have short- and long-term psychophysical complications and that even persons who have willingly agreed to undergo the surgery (i.e., having given informed consent according to currently accepted medical standards) can nevertheless feel they have experienced a “genital mutilation.”

In the cases presented here, this feeling led the women to seek care at an outpatient clinic that usually receives migrants, refugees, second-generation or naturalized patients originating mainly from African countries having experienced FGM/C. These examples show that the above mentioned IUGA/AUGS flowchart—based on medical indication, intention to harm, and autonomous consent—may be simplistic, failing to capture the experiences and interpretations of affected individuals (Joint Writing Group of the International Urogynecological Association & the American Urogynecologic Society, 2022).

The three patients wanted to undergo FGCS but subsequently felt severely harmed mentally, sexually, and physically. They also expressed a feeling of betrayal both toward the surgeon and the procedure itself. We can assume in these cases that the surgeon did not want to harm his/her patient or be negligent and that he/she decreased the size of the patients’ labia according to standard practice and knowledge in agreement with the request and expectation of the patient. And yet, they still felt “mutilated.”

What is the difference, then, between a “cosmetic surgeon” and an “traditional cutter”? Given that the WHO includes fully “medicalized” FGM/C in its definition of the practice, and this is reflected in many laws, might trained healthcare providers be considered guilty of performing “FGM/C,” just so long as the assumed/inferred “reasons” for performing the procedure are not considered (sufficiently) “medical”? Difficulties in distinguishing “cultural” or “cosmetic” reasons from “medical” reasons for performing surgery of the vulva have been noted many times.

In Switzerland, for example, lawmakers drafting a 2012 law against FGM/C openly acknowledged the technical and anatomical similarities between so-called cultural genital modifications associated primarily with African immigrant communities, and “cosmetic” vulvar procedures carried out in Swiss clinics (Bader & Mottier, 2020). However, despite these similarities, only the latter procedures were considered consistent with “Swissness” (i.e., Swiss national identity), whereas the former were interpreted as “foreign”—a threat to the body politic. Seeking a rational criterion to distinguish them, it was alleged that Swiss-associated “cosmetic” surgeries “always” have a “medical reason” (even if only a vaguely “psychological” one), whereas it was not considered possible that there could be analogous “psychological” medical reasons for a migrant woman of African origins to seek to modify her vulva (Bader & Mottier, 2020).

Thus, in Switzerland, it remains the case that even underage girls of Swiss/European origin may legally undergo cosmetic genital surgeries for ill-defined “psychological” medical reasons (e.g., relief of distress), whereas even adult women of African origin cannot consent to “cultural” genital surgeries, regardless of the reason (Bader & Mottier, 2020).

If the IUGA/AUGS flowchart is to be improved and updated, the authors might think of a flowchart that distinguishes between procedures carried out non-voluntarily on minors versus consensually on adults; and it could cover all types of genital procedures, including intersex genital “normalization” surgeries and penile circumcision (Brussels Collaboration on Bodily Integrity, 2024). Scientific societies and the WHO might also consider studying the possibility of revising the current definition of FGM/C (Shahvisi et al., 2024). In doing so, it would be necessary to settle on a truly principled distinction between acceptable and unacceptable genital modifications that does not discriminate on the basis of race/ethnicity or sex/gender, nor simply reflect which set of practices happen to be associated with more dominant groups (Earp et al., 2024).

One proposal for such a distinction is that for all non-voluntary procedures, regardless of an individual’s sex characteristics or gender, the intervention must be urgently necessary on grounds of physical health, whereas for voluntary procedures, a wider range of reasons and values could factor into the decision, based on the explicit needs and preferences of the person concerned (Brussels Collaboration on Bodily Integrity, 2024). However, it would be necessary to ensure that the “voluntariness” of a procedure was determined by objective criteria having to do with standardized ways of assessing consent capacity (Buckler, 2023), or perhaps by adopting a uniform age-based distinction that applied equally to all individuals regardless of their racialized status or cultural background (as proposed by some authors to prevent, e.g., racial bias in assessments of voluntariness) (Townsend, 2023a, 2023b).

### Clinical Implications

The three patients from our case studies regretted the surgery and expressed that their pre-surgery vulva was “more normal and beautiful.” When we discussed in detail their psychosexual history, all three women disclosed a history of sexual violence. In Switzerland, a survey on 4495 women aged at least 16, revealed that one out of five had experienced sexual violence of some kind (Amnesty International, 2019). Such a history can impact self-esteem, psychosexual health, and the body image of patients seeking genital cosmetic surgery. These needs should be carefully and compassionately addressed before, during, and after such surgery if it is pursued.

Most women and girls requesting FGCS have physiologically normative external genitalia. Up to 87% are reassured through counseling, thus avoiding surgery (Shaw et al., 2022). Raising awareness among women and girls or any persons with a vulva questioning the “normality” of their genitalia is crucial, and can be achieved through showcasing the wide diversity of vulvas, for example through the Vulva Gallery (Atalanta, 2019), the Great Wall of Vulva (McCartney, 2024), All Vulvas are Beautiful campaign (“All Vulvas are Beautiful,” 2021), Labia Library (Women’s Health Victoria, 2011), and Vulva Museum To Go (“Vulvaversity,” n.d.).

Although many classifications of “hypertrophy of the labia minora” have been proposed, i.e., as a potential “medical reason” for performing labiaplasty, there is no consensus on the definition of inner labia hypertrophy in the literature (Boddy, 2020; Smarrito, 2017; Walden et al., 2024). The first classification of inner labia hypertrophy was published in 1992 by Felicio, a Brazilian plastic surgeon: if the distance between the distal margin of the inner labium base and the vaginal introitus was below 2 centimeters (cm), it was considered grade 1, whereas if it was above 2 cm (up to 4 cm) it was considered grade 2. Grade 3 was defined as 4–6 cm, and grade 4 as > 6 cm (Colaneri, 2018). A few other classifications were suggested, for example Motakef’s classification (2015), which takes into account the protruding tissue of the inner labia not covered by the outer labia (Colaneri, 2018). None of the classifications seems to encompass all aspects of “symptomatic hypertrophy” (Colaneri, 2018). The new classification by Colaneri takes into consideration both the labia size and its localization (below or above the clitoris) (Colaneri, 2018).

The word “hypertrophy” means that there is an increase in size/volume of some tissue or organ due to the enlargement of its component cells. However, healthy hypertrophic tissue does not necessarily need to be surgically or medically treated. After all, a mere increase in size or volume relative to some statistical-anatomical norm is not in itself indicative of a disease state. This is obvious in the case of “hypertrophic” breast or muscle tissues; the same point applies to the labia. Breast hypertrophy corrective surgery is generally considered to be medically indicated and is refunded when symptomatic. Symptoms may include human relational problems (embarrassment, sexual harassment, inadequacy or limitations in sport, sleeping or breathing) and physical symptoms such as neck, shoulder and back pain; hygiene problems; upper extremity neuropathy; postural change and problems with brassiere support and breast weight as well as spine deformity (Chidyllo, 2018; Perdikis et al., 2022).

Further studies on vulvar and labia diversity might better inform assessments about what constitutes “labial hypertrophy” in the future (Perelmuter, 2024). However, even then, an ethical distinction would need to be drawn between cases of labial hypertrophy that seem to warrant or even require medical or surgical intervention (i.e., “medically necessary” labiaplasties) (Earp et al., 2023b) from ones that do not require such intervention. Such clarifications could also help in better defining rules regarding health insurance refunding for medically necessary labiaplasty (Walden et al., 2024).

Once it has been established that there are clinical indications for labiaplasty, an appropriate technique must be chosen. Several different surgical techniques exist, which can be categorized in three groups: edge resection, wedge resection, and central resection (Kalampalikis, 2021). Edge resection consists in the resection of the most protruding part of the labia. The excision can either be straight or curved (S-shaped or W-shaped) to reduce scar contraction (Özer, 2018). Wedge resection is the most popular surgical approach. It was developed to preserve the shape and coloration of the labium, and to prevent loss of sensation. The location of the wedge resection is adjusted according to the most protuberant part of the inner labia. Wedge resection’s main advantage is that the labia cannot be over-reduced (Özer, 2018). Central resection is a technique in which the outlines of the labium are preserved. It also allows preservation of the natural coloration of the inner labia (Özer, 2018).

We were unfortunately unable to obtain the surgical reports from the first and the second patients, but they probably underwent a wedge resection. As for the third patient, her surgical report stated that the surgeon performed a wedge resection.

Cosmetic genital surgery is very rarely taught and the number of defects post-labiaplasty due to inexperience, underestimation of the scope of the surgery, and lack of anatomical knowledge can be significant (Gress, 2021). In addition, labiaplasty is rarely primarily motivated by physical complaints such as pain, infections, discomfort, or hygiene difficulties. Instead, women often seek the procedure due to dissatisfaction with the appearance of their vulva and/or associated psychosexual dissatisfaction (Özer, 2018; Zwier, 2014). Because of these reasons, doctors should not underestimate the scope or severity of the possible physical, psychological, and sexual issues associated with a surgery that could initially appear to be simple (see Walden et al., 2024, for details and caveats).

Little is known about the actual prevalence of sexual violence history or child sexual abuse in the general population that also applies to those who request cosmetic genital surgeries—also because surgeons do not always ask the question. Body dysmorphic disorder, depression, anxiety, and personality disorder have been found in 4 to 50% of those who undergo cosmetic surgery (Bascarane et al., 2021), and all these latter mental health conditions can be associated with past sexual abuse (Constantian, 2018). A study comparing women seeking labiaplasty with a control group did not find a more frequent history of neglect or abuse during childhood (Veale et al., 2014). Labiaplasty might be sought for other forms of anxiety, trauma, psychosexual issues, or low self-esteem, including after sexual violence, and thus counseling and non-surgical care might be more appropriate, and should at least precede the surgery (Vieira-Baptista et al., 2018). If offered, surgery should be performed by a trained surgeon or team able to deal with the potential surgical and psychophysical complications.

Psychosexual vulnerabilities have to be appropriately screened. Patients should receive proper care. Indeed, if the patient asking for a labiaplasty suffers from chronic vulvar pain, PTSD after sexual violence, or from other vulvar conditions (e.g., Lichen Sclerosus), the right treatment might not be, or might not only be, FGCS.

Tables 2 and 3 summarize the available guidelines on FGCS of ObGyn and plastic surgery societies. It is important to highlight that while no ObGyn societies recommend FGCS, the American Society of Plastic Surgeons describes a high satisfaction rate of 90% and states that FGCS usually relieves symptoms in symptomatic patients (American Society of Plastic Surgeons, 2020b).

**Table 2:** Summary of available guidelines in English of ObGyn and plastic surgery societies

Society	General recommendations	Adolescents	Advertisement	Other	Text on FGM/C
SOGC (Society of Obstetricians and Gynaecologists, Canada)	FGCS for non-medical reasons cannot be accepted. Gynecologists and cosmetic surgeons should educate women asking for FGCS about their anatomy and the wide variations of normal vulvas, as well as physiological changes over the lifespan (Shaw et al., 2022)	16 years old threshold. For teens, additional expertise in counseling is required (Shaw et al., 2013)	Caution should be applied in advertising FGCS, to ensure such advertising is factual and not misleading (Shaw et al., 2022)	Surgeons should also assess patients’ sexual and psychological well-being, such as the absence of body dysmorphic disorder (Shaw et al., 2022)	Performing FGM/C in Canada is a criminal offense. FGM/C is considered as aggravated assault (up to 14 years of imprisonment) (“Ontario Human Rights Commission,” n.d.)
RCOG (Royal College of Obstetricians and Gynaecologists, UK)	RCOG warns doctors performing FGCS that they are operating without a clear evidence base: there is no data on the efficacy of FGCS on physical discomfort and pain, nor on sexual and cosmetic satisfaction (RCOG Ethics Committee, 2013)	FGCS should not be performed on persons below 18 years of age, due to physiological changes of the vulva during puberty (RCOG Ethics Committee, 2013)	Due to the lack of data on FGCS’s efficacy, advertising those surgeries is questionable (RCOG Ethics Committee, 2013)		Fine and/or up to 14 years of imprisonment (Female Genital Mutilation Act, 2003)
ACOG (American College of Obstetricians and Gynecologists, USA)	Patients should be informed of the lack of high quality data supporting the effectiveness of FGCS (ACOG, 2020)	FGCS in patients younger than 18 years old should only be considered for significant congenital malformation or persistent symptoms. The gynecologist should consider non-surgical alternatives (ACOG, 2020)	Advertisement must not be misleading and must be accurate. “Rebranding” existing surgical procedures and marketing them as new FGCS is misleading (ACOG, 2020)	Brazilian waxing (removal of most or all pubic hair) allows a better view of the vulva, which in turn can draw attention to asymmetries and differences in vulvas, leading to potential labiaplasties (ACOG, 2020)	FGM/C in case of patients younger than 18 years is punished by law (fine and/or imprisonment up to 10 years) (ACOG, 2020; House of Representatives of the United States of America, 2021)
RACGP (Royal Australian College of General Practitioners, Australia)	The patient should be examined by a doctor experienced in women’s health, and general practitioners should refer any patient having concerns about the appearance of their genitalia (Simonis, 2015)	For patients under age 18, RACGP advises not to operate, and a referral to a specialist adolescent gynecologist is required (Simonis, 2015)	Cosmetic surgery redefines the patient as a ‘consumer’, and advertising promotes a ‘product’. Advertising for FGCS could create dissatisfaction among women. Advertising suggests that FGCS are simple, and offer high satisfaction (Simonis, 2015)	Influence of trends, like form-fitting clothing, allowing others to guess the shape of the genital area, or G-strings, that cover a minimal portion of the genitals, could be taken to imply that female genitals should be small (Simonis, 2015)	If practicing FGM/C, penalties range from 7 to 21 years of imprisonment (Simonis, 2015)
NASPAG (North American Society for Pediatric & Adolescent Gynecology)	FGCS is not recommended. Affirming normalcy of the vulva during a physical exam empowers teens (Hillard, 2023)	Screen for body dysmorphic disorder. Counsel teenagers (Hillard, 2023)	Not specified	Not specified	Not specified
BritSPAG (British Society for Paediatric & Adolescent Gynaecology)	Labial development and growth may not be completed until early adulthood (British Society for Paediatric & Adolescent Gynaecology, 2013)	FGCS should not be performed on girls under the age of 18 years (British Society for Paediatric & Adolescent Gynaecology, 2013)	Not specified	Warning: the younger a girl has a labiaplasty, the higher the number of surgeries and the risk of scarring/loss of sensitivity (British Society for Paediatric & Adolescent Gynaecology, 2013)	Not specified
AmSPS (American Society of Plastic Surgeons)	FGCS usually relieves symptomatic patients. High satisfaction rate of 90% (American Society of Plastic Surgeons, 2020)	Not specified	Not specified	Not specified	Not specified
BAAPS (British Association of Aesthetic Plastic Surgeons, 2024)	Availability of FGCS in the UK is limited. Careful placement of the scar is crucial to prevent scar contraction (British Association of Aesthetic Plastic Surgeons, 2024)	Not specified	Not specified	Not specified	Not specified
AuSPS (Australian Society of Plastic Surgeons)	Labiaplasty may not be suitable for everyone. Complications (bleeding, asymmetry, wound infection, over or under correction, etc.) are mentioned. Revisional surgery is uncommon but can be necessary (Australian Society of Plastic Surgeons, 2024)	Not specified	Not specified	Not specified	Not specified

**Table 3:** Summary of the main messages in the available guidelines in English of ObGyn and plastic surgery societies

	FGCS Recommended?	FGM/C being a Criminal Offense?	Age Threshold for FGCS	Advertisement Permitted
SOGC	✕	✓ (jail up to 14 years)	16 yo	±
RCOG	✕	✓ (jail up to 14 years)	18 yo	±
ACOG	✕	✓ (fine or jail up to 10 years)	18 yo	±
RACGP	✕	✓ (jail up to 21 years)	18 yo	✕
AmSPS	✓	NS	NS	NS
BAAPS	±	NS	NS	NS
AuSPS	±	NS	NS	NS

It should be noted that the societies of pediatric and adolescent gynecology (for example, NASPAG (North American Society for Pediatric & Adolescent Gynecology) or BritSPAG (British Society for Paediatric & Adolescent Gynaecology)) do not recommend FGCS either

SOGC (Society of Obstetricians and Gynaecologists, Canada); RCOG (Royal College of Obstetricians and Gynaecologists, UK); ACOG (American College of Obstetricians and Gynecologists, USA); RACGP (Royal Australian College of General Practitioners, Australia); AmSPS (American Society of Plastic Surgeons); BAAPS (British Association of Aesthetic Plastic Surgeons); AuSPS (Australian Society of Plastic Surgeons); NS (not specified)

### Health Insurance Coverage

Relating to the above discussion, there is a lack of official recommendations in Switzerland (and also a lack of national and international consensus) on what is defined as “hypertrophic labia” or “symptomatic hypertrophy of the inner labia.” Because of this, reimbursements for labiaplasty are the decision of the “médecin conseil,” the doctor associated with the health insurance of the patient. Swiss health insurances do not cover the vast majority of labiaplasties as they do not treat a disease and are only rarely requested due to a physical injury following an accident (Bader, 2011; Société Suisse des médecins-conseils et médecins d’assurances, 2013). Medical justifications through written letters by the surgeon explaining why the surgery is indicated (e.g., symptoms, measurements, etc.) may increase the chances of reimbursement (Bader, 2011). The lack of written Swiss guidelines may result in difficulties in obtaining a refund for therapeutic surgeries on the labia according to standardized recommendations, such as those that are available for breast hypertrophy (Perdikis et al., 2022). In Switzerland, health insurance companies agree on potentially reimbursing breast reduction—meaning recognizing a medical reason—if the patient has a normal Body Mass Index (under 25 kg/m^2^) and if at least 500 g per breast can be removed—hence considering breast hypertrophy as the origin of the symptoms (Société Suisse des médecins-conseils et médecins d’assurances, 2013). On the contrary, in France, labiaplasty is the only cosmetic surgery that is 100% refunded (Sécurité Sociale, n.d.).

### Legal and Ethical Implications

FGM/C is a crime in Switzerland, as per Article 124 of the Swiss criminal code, which stipulates that “any person who mutilates the genitals of a female person, impairs their natural function seriously and permanently or damages them in some other way shall be liable to a custodial sentence not exceeding ten years or to a monetary penalty” (Swiss Confederation, 2011). Given that the women in our case studies all felt that they had, indeed, suffered genital mutilation, might they have recourse to the law in seeking to address their complaint? As it stands, the answer appears to be “no.” To the best of our knowledge, there have not been legal cases discussing this issue in Switzerland.

When drafting the Female Genital Mutilation Act, Swiss legislators sought to functionally exempt “FGCS” from the purview of the prohibition (similar to the UK and Australia; see, for example, Dustin, 2010). They concluded that “the prosecution authorities and the courts had to apply reason to prevent cosmetic surgery becoming subject to judicial proceedings” (Bader & Mottier, 2020). Although Article 124 of the Criminal Code does not explicitly exclude FGCS, Swiss legislators—conscious of the blurred line between the FGCS and FGM/C in determining which genital modifications might be considered as mutilations—decided that despite their “technical commonalities” a legislative double standard will be allowed (Bader & Mottier, 2020). According to some Swiss experts, moreover, few patients would be prepared, out of shame, to sue their surgeon and make their “failed” surgery public (Bader, 2011).

The problem of double standards in genital modification legislation has been raised by numerous scholars. These double standards do not only apply to FGM/C (associated with some racial, ethnic, cultural, or religious groups, often of a particular immigration status) versus FGCS (associated with different, typically more culturally dominant groups), but also to persons of different sex characteristics or genders in relation to the protection of their genital integrity (Brussels Collaboration on Bodily Integrity, 2019, 2024).

Anthropologist Fusaschi has recently proposed the term “gendered genital modifications” to encompass all forms of genital cutting and surgery across cultures and sex/gender systems, including not only FGM/C and FGCS, but also intersex “normalization” surgeries and even penile/male circumcision (Fusaschi, 2023). It then becomes possible to evaluate the practices, not in terms of the sex or gender of the affected person, which should be a morally irrelevant criterion, but along various ethically relevant dimensions including whether or to what extent the practice is medically necessary, how risky it is (including in relation to sexual functioning), and whether it is consensual.

Of course, it must be acknowledged that concepts of medical necessity and risk, as well as criteria for giving ethically valid consent to certain body modifications—including, but not limited to, genital modifications—are not a matter of universal consensus (Earp et al., 2023a). Nevertheless, it is increasingly argued that, *whatever* concepts and criteria are employed, they must be neutral as to race, sex, religion, or other protected characteristics, and applied to all persons equally without discrimination or bias (Ahmadu, 2017; Brussels Collaboration on Bodily Integrity, 2019, 2024; Earp, 2022; Townsend, 2020, 2023a, 2023b).

### Social Implications

Some authors have pointed out that advertisements of cosmetic surgeries on the vulva often present the norm for beauty as a prepubescent genital appearance. Surgery is then marketed as effective to achieve sexual pleasure and general well-being (Martin et al., 2017), to liberate and empower women (Martin et al., 2017), and as a quick and complication-free solution (Vieira-Baptista et al., 2018). Such advertisements, along with poor information about the diverse appearance of healthy vulvas, lead many women and girls to doubt of the normalcy of their genitalia which may then produce distress or embarrassment and sexual inhibition. Given the private status of this part of the anatomy, women and girls are often left alone to face their insecurities and decide to undergo surgery, sometimes even against the advice of their partner(s).

Beauty norms and standards available in the media lead to a growing pressure on women and girls (Chambers, 2022; Jeffreys, 2015). Many vulvas exhibited in pornographic movies underwent labiaplasties or were digitally modified to showcase “Barbie vulvas” (where inner labia have no protuberance beyond outer labia) (Alinsod, 2013). Scientific societies, in conversation with medical ethics experts and community members and patients, should establish clear criteria to decide whether and when labiaplasty is indicated, and, when it is, how and with what kind of psychosexual counseling or support it should be offered. There is also a need to offer written accessible documentation to persons who are thinking of labiaplasty. Surgeons should agree beforehand with the patient on the scope of the inner labia size reduction and be able to care for, support and/or refer their patients in case they are dissatisfied with the result, or are otherwise in distress or suffer from psychophysical complications.

### Conclusion

Even though not recommended by most scientific societies (see, e.g., ACOG, 2017), and with its legal status increasingly questioned by scholars (Berer, 2010; Dyer, 2019; Gaffney-Rhys, 2021), labiaplasty is currently treated as legal whereas FGM/C Type 2a is considered a crime on both minors and adults in several countries including Switzerland. The WHO launched its plan to “eradicate FGM/C” in 2008 (WHO, 2023); to date up to 18 African countries and 13 Western countries have enacted laws criminalizing FGM/C (Bootwala, 2023). Article 124 of the Swiss Criminal Code excludes FGCS (Conseil fédéral, 2020), which is not deemed illegal, even on minors. Some have claimed that the law punishes only genital modifications committed by or on extra-European migrants, without taking into consideration age, consent, or type of excision, whereas FGCS often responds to social beauty norms dictated by Western pornography and/or beauty industries (Bader, 2011).

While in the past, experts have maintained a discourse according to which FGCS and FGM/C have “nothing to do with each other” (Bader, 2011), many scholars and international societies have since warned of the inconsistencies and the empirical challenges surrounding the coexistence of FGCS and FGM/C (O’Neill et al., 2020). The three cases described in this paper further emphasize the blurred line between FGCS and FGM/C. Important medical, surgical, ethical, and legal questions should be tackled by scientific societies, legislators, and anti-FGM/C advocates, in collaboration with community members and patients, so that any harmful, unwanted, or non-voluntary genital modification is studied in relationship with other such phenomena. Concepts such as harm, reasons (religious, cultural, cosmetic), decision making, and consent should be applied equally and without bias: that is, regardless of the skin color of the individuals concerned, their racial, ethnic, or religious background, and the name given to the genital modification they underwent.

## Data Availability

Not applicable.

## Footnotes

1 Indeed, in some cases there may be no universal or objective criterion for counting something as a medical reason. This is because whether something is perceived to be a medical reason in some context is often a matter of contingent social construction.
